# Do non-pathogenic variants of DNA mismatch repair genes modify neurofibroma load in neurofibromatosis type 1?

**DOI:** 10.1007/s00381-021-05436-w

**Published:** 2022-01-08

**Authors:** Anja Harder

**Affiliations:** 1 Institute of Pathology, Medical Faculty, Martin Luther University Halle-Wittenberg, Halle (Saale), 06120 Germany; 2 Institute of Neuropathology, University Hospital Münster, Münster, Germany; 3 Faculty of Health Sciences, Joint Faculty, Potsdam, Germany

**Keywords:** Mismatch repair, MMR, Neurofibromatosis type 1, NF1, Modifier, Neurofibroma

## Abstract

Non-pathogenic mismatch repair (MMR) gene variants can be associated with decreased MMR capacity in several settings. Due to an increased mutation rate, reduced MMR capacity leads to accumulation of somatic sequence changes in tumour suppressor genes such as in the *neurofibromatosis type 1* (*NF1*) gene. Patients with autosomal dominant NF1 typically develop neurofibromas ranging from single to thousands. Concerning the number of neurofibromas NF1 patients face a situation that is still not predictable. A few studies suggested that germline non-pathogenic MMR gene variants modify the number of neurofibromas in NF1 and by this mechanism may promote the extent of neurofibroma manifestation. This review represents first evidence that specific non-pathogenic single nucleotide variants of MMR genes act as a modifier of neurofibroma manifestation in NF1, highlighting *MSH2* re4987188 as the best analysed non-pathogenic variant so far. In summary, besides *MSH2* promotor methylation, specific non-pathogenic germline *MSH2* variants are associated with the extent of neurofibroma manifestation. Those variants can serve as a biomarker to facilitate better mentoring of NF1 patients at risk.

**Supplementary information:**

The online version contains supplementary material available at 10.1007/s00381-021-05436-w.

## Introduction

Among DNA repair pathways, intact mismatch repair (MMR) is responsible for reparation of spontaneous DNA replication errors such as single-base-pair mismatches and small insertions or deletions in repetitive sequences that escape proofreading activity of DNA polymerase. In humans, mainly mutS homolog 2, mutS homolog 3, mutS homolog 6, mutL homolog 1, and post-meiotic segregation increased 2 (MSH2, MSH3, MSH6, MLH1, PMS2) interact to ensure proper repair and to maintain genomic stability. Defective MMR is associated with up to 1000‐fold increased mutation rate [1]. Additional functions of MMR were uncovered such as involvement in immunoglobulin gene hypermutation and autoimmune disorders, DNA damage surveillance, transcription coupled repair, genetic recombination and meiosis [2, 3].

Germline monoallelic or biallelic pathogenic variants (mutations) in MMR genes are involved in cancer predisposition syndromes, such as Lynch syndrome (LS) and constitutional mismatch repair deficiency (CMMRD) [4–7]. The type of sequence change in one of the MMR genes defines penetrance. More severe phenotypes result from *MLH1* and *MSH2* pathogenic variants in both LS and CMMRD [7, 8]. According to Knudson’s hypothesis, in autosomal dominant LS, the wild-type allele of either *MLH1*, *MSH2*, *MSH6*, or *PMS2* is inactivated in tumours due to small sequence changes. A resulting complete gene inactivation leads to a complete loss of MMR pathway functions with subsequent hypermutability which is termed mutator phenotype. MMR deficiency induces specifically frameshifts due to slipped mispairing in susceptible repetitive mononucleotides, dinucleotides, trinucleotides, or tetranucleotides (microsatellites) and is leading to microsatellite instability (MSI) in tumours. In colon cancer cells, the percentage of instable microsatellites is used to categorize somatic MSI into low MSI (MSI-L, 10–30%), high MSI (MSI-H, ≥ 40%), or stable MSI (MSS, 0%), although only MSH-H leads to a complete MMR deficiency [9, 10]. It is important to use a defined marker panel that is reliably associated with MMR deficiency [11]. The type of pathogenic variants in repetitive sequences is associated with the extent of MSI and predicts clinical presentation, choice of conventional therapy, and outcome: Mononucleotide sequence changes are typically associated with most MSI-H tumours (often *MLH1*, *MSH2*, and *MSH6* mutated) which are sensitive to 5-fluorodeoxyuracil. Dinucleotide sequence changes are mostly associated with MSI-L tumours. Tetranucleotide sequence alterations are a result of mutations in *MSH3* gene (described as ‘elevated microsatellite alterations at selected tetranucleotide repeats’, EMAST) being comparable to MSI-L and sensitive to poly-ADP-ribose polymerase 1 (PARP1) inhibitors [9].

In autosomal recessive CMMRD, patients suffer from childhood haematological malignancies, brain cancer, early-onset colon cancer, and other malignancies. Patients typically show clinical features of LS and of rasopathies including neurofibromatosis type 1 (NF1). NF1-like tumours (e.g. neurofibromas) are explained by somatic *NF1* pathogenic variants induced by MMR deficiency [7, 12]. Neurofibroma-like tumours were also shown in zebrafish due to MMR deficiency [13]. Although in LS pathogenesis of tumours is clearly related to MSI on the somatic level, causative genetic events for development of different tumours in CMMRD are unclear.

In general, cells with MMR deficiency experience an enormous increase of sequence alterations which explains development of both multiple cancers in tumour predisposition syndromes (such as LS and CMMRD) and in sporadic tumours. Cancer type, disease onset, and multiplicity of tumours depend on the MMR gene involved in germline. In sporadic neoplasia, MMR deficiency occurs only at the somatic level, being caused by *MLH1* hypermethylation as described in endometrial and sporadic (not associated with LS) colorectal cancer [14].

Due to recent awareness the term ‘mutation’ is to be avoided and standard terminology suggests a five-tier classification system of sequence variants into ‘pathogenic’, ‘likely pathogenic’, ‘uncertain significance’, ‘likely benign’, and ‘benign’ in Mendelian disorders [15]. Historically, a mutation was interpreted as a permanent sequence and polymorphism as a variant with a frequency above 1%. To bring new guidelines and anterior literature together within this review, we call traditionally termed mutations in literature ‘pathogenic variants’ and all others such as reported polymorphisms and rare variants ‘non-pathogenic variants’. Since the term pathogenicity is complex, this approach may not be precise but helps to integrate whole literature. Besides pathogenic variants in diseases as described above, also previously considered non-pathogenic MMR variants have been associated with disease modifications such *MSH3* rs26279, *MLH1* rs1800734, or other *MLH1*, *MSH3*, and *DHFR* non-pathogenic variants [16–21]. To conclude, some missense non-pathogenic variants of MMR genes have already been extensively studied and demonstrated to produce mutator phenotypes in vitro, and therefore have been proposed to predispose humans to disease [22].

### Mismatch repair and neurofibromatosis type 1

The autosomal dominant tumour predisposition syndrome neurofibromatosis type 1 (NF1; MIM# 162200) results from germline pathogenic variants (mutations) of the tumour suppressor gene *NF1* on 17q11.2. NF1 is characterized by a variety of symptoms with development of multiple neurofibromas as a hallmark. The *NF1* gene appears to be prone to pathogenic variants and may be specifically affected by MMR deficiency: The *NF1* mutation rate is about 10-fold higher than described for other genes which has been attributed to the large size of the gene (350k bp, 61 exons). De novo germline pathogenic variants occur in about 50% of cases, and approximately 80% of those are of paternal origin. Recent studies demonstrated that mutation rates rise with increasing paternal age which also explains findings in NF1 very well [23, 24]. In general, mutation rate depends on transcription-coupled repair and the genomic context in dependence on epigenetic modifications and conserved regions: hypermutable methylated CpG dinucleotides (CpG effect) and DNase I hypersensitive sites increase mutation rate in specific regions [23, 25]. In *NF1*, the majority of small deletions and insertions occurs in homo-nucleotide tracts and substitutions are strongly associated with homo-nucleotide repeats and CpG/CpNpG motifs indicating a specific role of methylation [26]. Like other tumour suppressor genes, *NF1* covers both CpG islands, methylated nucleotides, and repeat sequences. The high mutation rate of the *NF1* gene might also be explained by gene conversion due to pseudogenes and by the high number of exons, as compared to other genes. Defective MMR also prefers specific sequences to mutate at faster rates than others such as short pair repetitive sequences, which is why it may be of interest to elucidate if the *NF1* gene covers more of those sequences than other genes [27].

MMR deficiency contributes to a tissue specific tumour development due to cellular characteristics that encompass a high proliferation rate (such as gastrointestinal cells) or rapid acceleration/deceleration of proliferation (such as endometrium), a dependence on specific mutator targets, functions for immune surveillance, apoptosis, toxin exposure, and mode of inheritance [28]. Concerning development of neurofibromas that arise from skin-derived progenitor cells, more than one of these cellular characteristics is fulfilled for NF1. Accumulation of second *NF1* hits occurring in rapidly proliferating cells (prone to accelerated *NF1* mutation rate) may explain increased  tumour load arising from a decreased MMR capacity in NF1.

Whether source of increased somatic mutation rates is different compared to germline mutation rates in NF1 still remains unknown.

In neurofibromatosis patients, benign neurofibromas develop when the *NF1* gene is completely inactivated. But besides the first/germline and a second/somatic hit of the wildtype *NF1* allele, other factors modify tumour development [29].

Somatic *NF1* pathogenic mutations in tumours occur in several settings: In NF1 patients, a second *NF1* hit occurs in *NF1* associated nerve sheath tumours besides a germline *NF1* pathogenic variant (first hit) and leads to biallelic inactivation of *NF1* following the Knudson two-hit hypothesis. In sporadic malignant tumours such as melanomas, lung and breast cancer, and many others, a novel second *NF1* hit occurs. In this setting, the *NF1* hit act solely or together with other tumour suppressor gene alterations and is interpreted to be a driver mutation and even  predict resistance to therapy [30, 31]. In CMMRD associated tumours and other features, such café-au-lait spots, a second double *NF1* hit leads to a biallelic *NF1* inactivation which may be difficult to distinguish from mosaic or segmental NF1 [12].

In NF1, malignant peripheral nerve sheath tumours and benign neurofibromas differ in their mutational spectrum of the second hit. Neurofibromas show an increased proportion of small sequence changes [32]. This indicates that different mutational mechanisms are involved in tumorigenesis and suggest that a reduced MMR capacity probably due to minor alterations in MMR genes may influence neurofibroma development. One should necessarily not mix up that constitutional MMR pathogenic gene variants such as in CMMRD are associated with a distinct tumour syndrome that only focally resembles NF1 [33].

Investigating the function and mutational spectrum of *NF1* consistently leads to a reflection of genotype-phenotype-correlations. Several single correlations have already emerged, but the question why patients may exhibit only very few or thousands of neurofibromas is not yet clarified. Only microdeletion NF1 patients are known to suffer from a higher tumour burden and more severe phenotype. Nevertheless, patients without microdeletions may also suffer from a multiplicity of neurofibromas.

### MMR capacity and manifestation of cutaneous neurofibromas in NF1

**Fig. 1:**
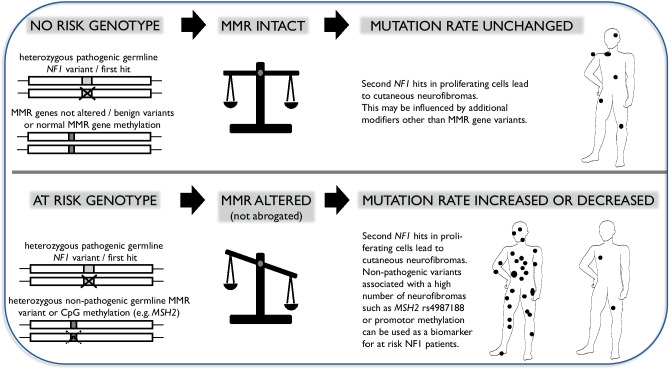
How MMR capacity may impact somatic *NF1* mutation rate and may modulate neurofibroma manifestation in proliferating cells.

Modifiers of the NF1 phenotype have been discussed and proposed [34–37]. In 2003, Wiest and co-workers suggested that MMR genes may be modifiers since they lead to accumulation of second hits in NF1 [38]. Further evidence came from a *mhl1*-deficient mouse model [39]. In 2009, non-pathogenic variants in MMR genes were shown to be associated with neurofibroma load, possibly explaining variability in the number of neurofibromas and indicating a role of a biomarker to identify patients at risk for a high neurofibroma load (Fig. 1) [40].

CMMRD patients show a distinct reduction of MMR activity that can be measured even by in vitro assays in blood lymphocytes to diagnose the disease with 100% specificity and sensitivity [12, 41, 42]. In contrast, the key mechanism in NF1 patients might be a reduced but not absent MMR capacity. In the study by Shuen and co-workers, MMR activity in lymphoblastoid cell lines of patients with NF1 was comparable to controls [42]. Similar results were obtained by another study that did not detected MSI in lymphocytes of patients with de novo *NF1* mutations [43]. This finding is not unexpected because in NF1, the reduced MMR activity should be mild and only be associated with a specific NF1 phenotype, namely the one with a higher frequency of second *NF1* hits and subsequent biallelic inactivation of *NF1* on the somatic level. Such a mild event unfortunately implicates that every germline MMR variant in NF1 needs to be examined in detail and for segregation. Interestingly, a general finding fits very well to this hypothesis: a small to moderate reduction of the DNA repair capacity was proposed to affect cancer predisposition and to be more difficult to prove (Mohrenweiser et al., 2003).

In 2014, a genome-wide screen identified MMR gene transcripts associated with the number of café-au-lait macules (CALM) and identified MMR non-pathogenic single nucleotide variants associated with CALM count [44]. Since CALM in NF1 also develop from biallelic *NF1* inactivation, this study supports the above hypothesis. In a previous study, epigenetic MMR gene events were associated with neurofibroma burden: *MSH2* promoter methylation was observed in NF1 cases (*n* = 79) compared to controls (*n* = 39). Additionally, a significantly increased methylation of two *MSH2* CpG dinucleotides was seen NF1 patients with higher neurofibroma count [40]. Germline epimutations of *MSH2* that have been described in HNPCC support the significance of this so-far unique finding in NF1 [45].

There is no doubt that the *NF1* gene is a target of reduced MMR activity (Wang 2003). A study investigating the spectrum of somatic pathogenic variants in Schwann cells from 38 neurofibromas suggested slightly reduced DNA repair efficiency as mechanism (Maertens 2006). The authors described that the spectrum of somatic non-pathogenic variants differs between microdeletion and non-microdeletion NF1 patients, and they suggested that the high frequency of somatic frameshifts can result from reduced DNA repair efficiency due to aging. Interestingly, the authors detected  *MSH2* variant re4987188 (G322D) to be associated with a higher neurofibroma burden and reduced MMR activity in other experiments.

Concerning MSI that would normally arise from a reduced MMR capacity, data from studies of NF1 associated neurofibroma are conflicting. Two studies detected MSI in neurofibromas, whereas others did not (for reference see Table 1). As mentioned above, due to a proposed milder effect of MMR on neurofibroma manifestation, MSI may not be detected in neurofibromas although non-pathogenic MMR gene variants influence severity of tumour manifestation. To underline this, e.g., *MSH2* rs2303428 TC + CC genotype was shown to predict prognosis and adjuvant chemotherapy benefit in non-cardia gastric patients but was not associated with MSI suggesting lower penetrance and probably also MSI-independent mechanisms [46].

**Table 1:** Studies investigating microsatellite instability in NF1 associated neurofibromas

**Markers used for MSI analysis**	**Number of neurofibromas/all neurofibromas investigated**	**References**
D17S250, D5S107, DISIO4, D8S87, DJJS9OS	**8/16**	Ottini et al. [47]
IVS27AAAT2.1, IVS27AC28.4, IVS27AC33.1, IVS38GT53.0, D17S250	0/60	Serra et al. [48]
BAT25, BAT26, BAT40, D2S123, D5S346, D17S250	0/20	Luijten et al. [49]
MYCL, BAT26, D2S123, D17S250, APC D18S58	0/70	Upadhyaya et al. [50]
MYCL, BAT26, D2S123, D17S250, APC D18S58	0/n (multiple tumours in 1 patient)	Stewart et al. [51]
MYCL, BAT26, D2S123, D17S250, APC, D18S58	0/n (multiple tumours in 1 patient)	Spurlock et al. [52]
D13S153, D5S406, D5S107, BAT26, ACTC, D2S123, D17S250, BAT-25, BAT-40.4, D5S346	**21/89** (of 3 NF1-patients with > 550 neurofibromas)	Thomas et al. [53]

From a different point of view, in 2007, Garza and co-workers proposed that genomic instability influences tumour progression in NF1 and disease severity, because they discovered a mild mutator phenotype in tumours of a mouse model targeting *Nf1* and *p53* in cis. Although in this model *TP53* haploinsufficiency is the basic cause for limited repair capacity of lesions, genomic instability seems to play a role in NF1 associated benign and malignant lesions.

## Material and methods

A comprehensive literature and database search was undertaken to identify those studies reporting single-nucleotide non-pathogenic variants of MMR genes that potentially affected somatic *NF1* mutation frequency and therefore possessed the ability to modify neurofibroma load in patients.

## Results and discussion

### Non-pathogenic MMR gene variants and neurofibroma load in NF1

We will report only those studies that investigated single-nucleotide non-pathogenic variants of MMR genes that potentially affect somatic *NF1* mutation frequency and therefore possess the ability to modify neurofibroma load in patients. To date, only three studies investigated the association of specific non-pathogenic variants and load of neurofibroma (Table 2). In 2009, our group hypothesized that MMR genes may be associated with a NF1 phenotype of a high versus a low neurofibroma burden and demonstrated that the extent of methylation of two CpG dinucleotides of *MSH2* promotor in leukocytes correlated with an increased number of neurofibromas [40]. In that study, we also fully sequenced MMR genes facilitating re-analysis of single nucleotide variants of *MSH2*, *MSH6*, *MLH1*, and *PMS2*. Against the background of novel studies, we compiled our data and compared non-pathogenic variants between NF1 patients with a high (*n* = 38) and those with a low neurofibroma burden (*n* = 41) which we had previously defined as major and minor disease manifestation. Overall, we detected 10 non-pathogenic variants in *MSH2*, 1 non-pathogenic variant in *MLH1*, and 10 non-pathogenic variants in *PMS2*, and, as expected, did not identify constitutional pathogenic MMR gene variants (see supplementary material) which is supported by other studies [53, 54, 43].

**Table 2:** Studies investigating relationship between mismatch repair (MMR) activity and tumour manifestation in neurofibromatosis type 1 (NF1) with specific regard to number of neurofibroma and beyond investigating microsatellite instability (MSI)

Study	Study size and patients	Main message
Wang et al. [43]	20 NF1 patients, 15 human MSI cancer lines, *mlh1*-deficient mouse embryonic fibroblasts, 5 primary tumours	*NF1* is a target of MMR deficient (non-NF1) cell type. No MMR gene mutations or MSI in lymphocytes of patients with de novo NF1 mutations.
Maertens et al. [54]	48 dermal neurofibromas from 9 unrelated NF1 patients, 15 additional NF1 patients for variant analysis, controls	Observed MMR gene variants p.I219V (*MLH1*), p.G322D (*MSH2*), and p.G39E (*MSH6*) in NF1 cohort. Variant p.G322D led to reduced MMR activity and was associated with increased load of neurofibromas. Hypothesized that mutation signature (many frame-shifts) of neurofibromas reflects reduced DNA repair efficiency as a trigger for *NF1* somatic inactivation.
Titze et al. [40]	79 NF1 patients, 79 controls	Methylation grade of 2 CpGs of *MSH2* promotor correlated with a high burden of neurofibromas. *MLH1*, *MSH6*, and *PMS2* were not methylated in leukocytes of NF1 patients. A higher rate of methylation in NF1 patients occurs compared to controls. Hypothesis that methylation induced variability of *MSH2* gene expression leads to variable mismatch repair capacity and modification of tumour load.

Those non-pathogenic variants are now reviewed that were detected in NF1 patients and were analysed with respect to neurofibroma burden and/or were found in other studies which helps to underline significance. Since meanwhile a plenty of data on non-pathogenic MMR variants are available, we only focussed on those that can be evaluated as a risk factor or as modifier.

### Non-pathogenic *MSH2* variants and neurofibroma manifestation

There is strong evidence that the common non-pathogenic missense variant re4987188 (G322D) acts as a modifier. The residue is highly conserved between species, and an equivalent alteration was investigated in yeast [55]. It was shown to act damaging due to disruption of splicing enhancers by bioinformatic algorithms and to moderately decrease MMR efficiency as well as to produce a dominant mutator effect in yeast [22, 56, 57]. In older studies, allele frequencies did not give rise to assume that this non-pathogenic variant could be pathogenic, and interpretations varied from deleterious to benign [58–62]. Re4987188 was also in silico proposed to be non-pathogenic (Thompson et al., 2013). However, in our and another study, it was associated with a higher neurofibroma burden [54]. Maertens and co-workers additionally showed an effect on reduction of MMR activity. In more recent studies, re4987188 was also associated with increased risks to vestibular schwannoma (odds ratio (OR) 1.67), recurrent colon cancer, lung cancer (OR 1.29), and breast cancer (OR 10.61) clearly attributed to the A allele in most studies [16, 63–65]. From current data, it can therefore be concluded that the A allele, being detected only in NF1 patients with a high tumour burden, serves as a disease modifier towards an increased neurofibroma load. *MSH2* re4987188 therefore needs to be evaluated in larger studies since it could be extremely useful for clinical risk assessment in NF1.

According to our analysis, two other non-pathogenic variants are of potential interest: *MSH2* rs771126636 and rs63750810 are both associated with a high neurofibroma burden (see supplementary material). Both variants have not been referenced in literature so far. Genotype C/A of *MSH2* missense variant rs771126636 is a still undescribed genotype in NF1 patients with a high tumour load. *MSH2* rs63750810 allele G is very rare and solely occurred in a patient with a high tumour load in our study. Since both non-pathogenic variants have not yet described elsewhere, it is very difficult to assess their relevance. Given their increased frequency in the cohort with a high neurofibroma load, larger studies as well as segregation and experimental studies might provide additional useful insights.

Other non-pathogenic *MSH2* variants such as rs17224360, rs2303426, rs61756467, and rs2303428 are also of potential interest, although associations are not as strong. For example, *MSH2* rs2303426 was found to be significantly associated with a higher risk for gallbladder cancer (OR 1.83) but neither differences between allele frequency or heterozygosity concerning risk of tumour burden in NF1 were detected in our study nor specific risk associations evoked from several larger cancer studies [66]. The same applies to intronic rs3732183 which may influence cis-acting regulatory elements and enhance *MSH2* expression: It was described to be a prognostic marker for patients with colorectal cancer (GG genotype predicted better survival), significantly associated with a better response to oxaliplatin-based chemotherapy of patients with advanced colorectal adenocarcinoma (OR for A/G + G/G genotype of 5.3). Furthermore, G/G genotype was associated with a lower risk of relapse in oral squamous cell carcinoma patients, but no specific effect was seen in our NF1 study [67–69]. Interestingly, data on *MSH2* rs3732183 remain conflicting as shown in a meta-analysis that demonstrated that this non-pathogenic variant had no major influence on overall cancer risk, but when stratified by cancer types the risk for non-Hodgkin’s lymphomas was increased which may indicate different roles in different cancer types [70].

Another non-pathogenic variant (rs2303428) of an acceptor splice site in intron 12 within a polypyrimidine tract was detected in our NF1 cohort but did neither demonstrate differences in heterozygosity or genotype concerning neurofibroma load. Nevertheless, it was associated with partial exon skipping 13 in splicing assays and with modification of prognosis of cancer or therapy side effects as well as benefit of chemotherapy of multiple other tumour diseases (reviewed in online resources) [71]. Those characteristics convincingly favour the hypothesis of a tumour-type dependent effect of non-pathogenic MMR variants.

To conclude, there is evidence that both methylation and specific non-pathogenic variants of *MSH2* serve as a disease modifier for neurofibroma load in NF1.

### Non-pathogenic *MLH1* and *PMS2* variants and neurofibroma manifestation

In *MLH1*, frequent non-pathogenic missense variant rs1799977 (I219Val) was shown to be associated with increased risk of sporadic colorectal cancer but unrelated to MSI: genotypes A/G and G/Gs were associated with odds ratios from 1.89 to 3.05 indicating that G allele carriers were at greater risk [72]. Those carriers had a decreased probability of vascular invasion, distant metastasis, re-occurrence, and improved outcome. The authors interpreted that this non-pathogenic variant only modulates but not abrogates MMR activity. In our study, this non-pathogenic variant was associated with a discrete difference and no specific genotypes or alleles in dependence to tumour load were seen. Maertens and co-workers also identified s1799977 in their NF1 study (allele frequency of controls (74/184, 40%)) and of NF1 patients (6/18; 33%) with an insignificant difference, but only in patients with a severe phenotype and without microdeletions [54]. Thus, although more than 40 studies analysed rs1799977 among others in cancer, only the two above mentioned studies exist so far in NF1 patients. From these few data, an influence on neurofibroma development might be inferred, but is still inconclusive.

In *PMS2,* a few non-pathogenic variants were described to be associated with a decreased risk. A allele of rs3735295, which we detected in our NF1 cohort, was shown to associate with a reduced risk for papillary thyroid carcinoma in Belarusian children after exposure to fallout from the Chernobyl power plant accident [73]. Unfortunately, although we observed differences in heterozygosity, we did not detect a specific genotype concerning neurofibroma burden. Whereas only rs112796669 showed predominant A allele in patients with a high tumour burden, two other variants of *PMS2*, rs12532895 and rs1805326, were linked to a low tumour burden in our study (see online resources). Rs12532895 has already been detected in a cohort of colon cancer patients [74]. Thus, non-pathogenic variants in *PMS2* were more often associated with a very mild NF1 phenotype. Since co-segregation studies of these rare non-pathogenic variants are missing, their association with neurofibroma burden is not confirmed and further experiments are needed. Testing larger cohorts as well as genome wide association studies of well characterized, sex, age-, and variant type-matched groups seem to be necessary to prove further associations of non-pathogenic MMR sequence variants and the extent of tumour burden in NF1.

## Conclusions

Independent of constitutional pathogenic variants (mutations), specific non-pathogenic MMR gene variants are associated with decreased MMR capacity. Reduced MMR capacity can lead to accumulation of somatic *NF1* sequence alterations and promote manifestation of NF1 associated cutaneous neurofibromas.

Already in childhood, NF1 patients typically develop neurofibromas within a range from single to thousands which cannot be predicted by the pathogenic germline *NF1* variant instead of microdeletion cases. Some studies suggested that non-pathogenic MMR gene variants modify the number of neurofibromas in NF1 as they similarly act as a modifier in other diseases. From the data reviewed here, there is strong evidence that specific non-pathogenic single nucleotide variants of MMR genes act in the proposed way. *MSH2* re4987188 is one of best analysed so far and is associated with a high burden of neurofibromas. Other promising non-pathogenic variants being associated with a high or low number of neurofibromas have been detected, but data sets are limited.

In summary, non-pathogenic germline *MSH2* variants and *MSH2* promotor methylation was shown to be associated with the extent of neurofibroma manifestation in NF1 patients (without microdeletions) indicating an influence on MMR activity. Accessible sequencing data should be used to establish more of those associations facilitating a far-sighted mentoring of our neurofibromatosis patients in future.

## Supplementary information

Below is the link to the electronic supplementary material.

Supplementary file1 (PDF 729 KB) Overview of non-pathogenic germline MMR gene variants detected in Neurofbromatosis type 1 (NF1) patients of the study (A). Selected data on non-pathogenic MMR gene variants from the study that demonstrated a difference in heterozygosity or a rare genotype comparing Neurofibromatosis type 1 (NF1) groups of low and high neurofibroma load (B).

## Data Availability

Additional data on MMR gene analysis is provided by Anja Harder.
