# Supplementary material for: Do non-pathogenic variants of DNA mismatch repair genes modify neurofibroma load in neurofibromatosis type 1?
Source: Childs Nerv Syst. 2022 Jan 8;38(4):705–13. doi: 10.1007/s00381-021-05436-w (PMC8940751; doi:10.1007/s00381-021-05436-w)
Supplement: Supplementary file 1 — Supplementary file1 (PDF 729 KB) Overview of non-pathogenic germline MMR gene variants detected in Neurofbromatosis type 1 (NF1) patients of the study (A). Selected data on non-pathogenic MMR gene variants from the study that demonstrated a difference in heterozygosity or a rare genotype comparing Neurofibromatosis type 1 (NF1) groups of low and high neurofibroma load (B). [file 381_2021_5436_MOESM1_ESM.pdf]

A) Overview of non-pathogenic germline MMR gene variants detected in Neurofbromatosis type 1 (NF1) patients of the study.

Variants detected by genomic Sanger sequencing (covering intron / exon boundaries) of *MSH2*, *MLH1*, and *PSM2* in NF1 patients with either a low or a high burden of cutaneous neurofibromas according to Titze et al., 2010. In this study the phenotype was defined by tumor number: ⩽ 60 tumors (mean 14,8 ± 13.3, n = 41, 15 males, 26 females) and at least > 100 tumors (range 112-2200, n = 38, 14 males and 24 females). Oligonucleotide sequences and analysis data are available on request from Anja Harder. *

Gene, variant ID	locali-zation	position	alleles	sequence position	codon change	amino acid change	ClinVar	H_{pop}	H_{NF1}	H_{low}	H_{high}
MSH2											
rs2303426	intron 1	Chr2:47630550	C/G	c.211+9C	-	-	likely benign	0.464	0.493	0.493	0.493
rs35898375	exon 1	Chr2:47635667	A/G	c.339G>A	AAG>AAA	Lys113Lys	benign / uncertain	0.010	0.012	0	0.026
rs4987188	exon 6	Chr2:47643457	A/G	c.965G>A	GGC>GAC	Gly322Asp	benign (missense)	0.033	0.049	0	0.091
rs771126636	exon 6	Chr.2:47643540	C/G/T(A)	c.1048C>A	CTC>ATC	Leu350Ile	uncertain (missense)	0	0.341	0.166	0.389
rs17224360	intron 6	Chr.2:47656871	C/T	c.1077-10T>C	-	-	benign	0.041	0.373	0.051	0.026
rs12998837	intron 9	Chr.2:47693788	A/T	c.1511-9A>T	-	-	benign	0.248	0.109	0.091	0.127
rs3732183	intron 10	Chr.2:47693959	G/A	c.1661+12G>A	-	-	benign	0.421	0.379	0.375	0.382
rs61756467	exon 11	Chr.2:47698179	A/G	c.1737A>G	AAA>AAG	Lys579Lys	likely benign	0.004	0.119	0.026	0
rs2303428	intron 12	Chr.2:47703500	T/C (A/G)	c.2006-6T>C	-	-	benign	0.160	0.194	0.185	0.204
rs63750810	exon 13	Chr.2:47703654	A/G	c.2154A>G	CAA>CAG	Gln718Gln	likely benign	0.002	0.012	0.185	0.204
MLH1											
rs1799977	exon 8	Chr.3:37053568	A/G (T)	c.655A>G	ATC>GTC	Ile219Leu	benign (missense)	0.439	0.416	0.442	0.385
PMS2											
rs890205609	5' exon 1	Chr.7:6048850	T/C	c.122A>C	-	-	unknown	0*	0.073	0.097	0.051
rs3735296	5' exon 1	Chr.7:6048804	C/G	c.-154C>G	-	-	benign	0.241	0.278	0.261	0.294
rs192027828	intron 1	Chr.7:6048618	G/C	c.23+10G>C	-	-	benign / likely benign	0.008	0.012	0	0.026
rs3735295	intron 1	Chr.7:6048556	G/A	c.23+72G>A	-	-	unknown	0.294	0.372	0.394	0.343
rs117831773	intron 3	Chr.7:6043495	T/C	c.251-72A>G	-	-	benign	0.080	0.043	0.058	0.020
rs12532895	exon 4	Chr.7:6043386	G/A (C/T)	c.288C>G	GCC>GCA	Ala95Ala	(likely) benign	0.064	0.042	0.017	0
rs79815075	intron 9	Chr.7:6029842	A/T	g.6029842	-	-	unknown	0.087	0.054	0.095	0.064
rs28699470	Intron 9	Chr.7:6029748	G/A	g.6029748	-	-	unknown	0.493	0.472	0.560	0.426
rs112796669	intron 9	Chr.7:6029700	G/A	g.6029700	-	-	unknown	0.002	0.036	0	0.054
rs1805326	intron 11	Chr.7:6022626	T/C	c.2007-4G>A	-	-	benign	0.273	0.027	0.58	0

H_{pop} represents heterozygosity of healthy population according to NCBI (1000 genomes, Europe subgroup) and calculated from appropriate allele frequencies. H_{NF1} represents heterozygosity of all NF1 patients investigated in this study, H_{low} and H_{high} denotes heterozygosity of NF1 patients with a low or high number of neurofibroma according to above defined criteria. Chromosome position is given according to GRCh37.p13. NA - not available. * according to ALFA, European subgroup, since 1000 genomes were not available.

B) Selected data on non-pathogenic MMR gene variants from the study that demonstrated a difference in heterozygosity or a rare genotype comparing Neurofibromatosis type 1 (NF1) groups of low and high neurofibroma load.

Gene, variant ID	Allele frequencies				Genotype (frequency, proportion of genotype / analysed individuals) in whole NF1 patient cohort.°°	Statistics and comments to NF1 cohort, references and general comments
	F_{pop} *	F_{NF1}	F_{low} #	F_{high} #		
MSH2						
rs35898375	G=0.995<br>A=0.005	G=0.994<br>A=0.006	G=1<br>A=0	G=0.987<br>A=0.013	G/G (0.99, 77/78)<br>A/G (0.01, 1/78): in one severely affected male patient with > 1000 neurofibromas.	Genotype A/G is also a rare in normal population.<br>No information from literature concerning association of genotypes and any risks available.
rs4987188	G=0.983<br>A=0.017	G=0.942<br>A=0.058	G=1<br>A=0	G=0.952<br>A=0.048	G/G (0.95, 57/60)<br>A/G (0.05, 3/60): only in severely affected NF1 patients.	Genotype A/G is also rare in normal population.<br>A/A strongly associated with increased risk for breast cancer (Smolarz et al. 2019), described as marker for risk of recurrence in HNPCC with higher frequencies of A/G and A/A alleles (Mik et al. 2017), associated with increased risk to lung cancer (OR 1.29), to vestibular schwannoma (OR 1.67) (Doherty et al. 2013; Rajaraman et al. 2010). Proposed as probably damaging (Doss and Sethumadhavan 2009) and shown to reduce MMR activity (Maertens et al. 2006).
rs771126636	C=1 **<br>T=0 **	C=0.942<br>A=0.058	C=0.5<br>A=0.166	C=0.734<br>A=0.265	C/C (0.57, 35/62)<br>C/A (0.43, 27/62): more common in NF1 patients with high tumour burden.	No data available for A allele.<br>Variant has not been described in literature so far. Variant with A allele has not been incorporated into database, thus we conclude that A/C genotype is very rare.
rs17224360	C=0.020<br>T=0.9791	C=0.019<br>T=0.037	C=0.026<br>T=0.974	C=0.013<br>T=0.987	T/T (0.96, 75/78)<br>T/C (0.04, 3/78).	Genotype frequency differs between the two NF1 groups.<br>Variant reported in some studies, but no risk association emerged for other cancers. No effect on splicing effects in-*vitro* (Tournier et al. 2008). Located in polypyrimidine tract of an acceptor and discussed to affect intron splicing (Roscigno et al. 1993).
rs12998837	A=0.854<br>T=0.145	A=0.942<br>T=0.058	A=0.952<br>T=0.048	A=0.932<br>T=0.068	A/A (0.88, 38/43)<br>A/T (0.12, 5/43).	A/T is rare in normal population.<br>Variant associated with increased risk to lung cancer among Non-Hispanic white smokers when stratified by MSH5 rs3131379 AG/AA genotype (Doherty et al. 2013).
rs61756467	A=0.998<br>G=0.002	A=0.994<br>G=0.006	A=0.987<br>G=0.013	A=1<br>G=0	A/A (0.99, 77/78)<br>A/G (0.01, 1/78): in one female, very mild affected patient and daughter.	A/G is rare in normal population.<br>Not analyzed in other cancer studies concerning risks. Aberrant splicing was not detected (Auclair et al. 2006).
rs2303428	T=0.913<br>C=0.088	T=0.891<br>C=0.109	T=0.897<br>C=0.103	T=0.885<br>C=0.115	T/T (0.78, 61/78)<br>T/C (0.22, 17/78)	No difference in genotypes between NF1 groups.<br>May predict prog-nosis and adjuvant chemotherapy benefit in non-cardia gastric cancer, correlated with risks of various cancers, including non-Hodgkin lymphoma, gastric, lung, endometrial and other cancer, predicted resectability in pancreatic cancer and was in-dependent prognostic factor in hepatocellular carcinoma, was related to the prognosis of ovarian cancer patients as well as was not associated with prognosis and/or chemoradiotherapy response in rectal cancer and head / neck squamous cell carcinoma (reviewed by Zhao et al., 2019) (Beiner et al. 2006; Doherty et al. 2013; Hishida et al. 2003; Jung et al. 2006; Paz-y-Mino et al. 2003; Paz-y-Mino et al. 2002; Si et al. 2019; Zhao et al. 2019). Not associated with cancer risks in other studies. Might serve as a predictive marker for hematologic side effects and response of melanoma treatment (Boeckmann et al. 2009).

MSH2, continued						
rs63750810	A=0.999<br>G=0.001	A=0.994<br>G=0.006	A=1<br>G=0	A=0.987<br>G=0.013	A/A (0.98, 77/78)<br>G/G (0, 0/78)<br>A/G (0.01, 1/77): in a patient with severe phenotype and in her father with >1000 neurofibromas.	G allele is very rare in normal population.<br>Variant has not been described in literature so far.
MLH1						
rs1799977	A=0.675<br>G=0.325	A=0.705<br>G=0.295	A=0.667<br>G=0.333	A=0.736<br>G=0.264	A/A (0.48, 32 /66)<br>A/G (0.44, 29/66<br>G/G (0.08, 5/66)	Discrete difference seen between NF1 groups.<br>Identified in another NF1 study only in patients with a severe phenotype (Maertens et al. 2006). Associated with increased risk for sporadic colorectal cancer (Nejda et al. 2009).
PMS2						
rs12532895	G=0.966<br>A=0.033	G=0.957<br>A=0.022	G=0.910<br>A=0.045	G=1.0<br>A=0	G/G (0.96, 66/69)<br>A/A (0.04, 3/69): in 3 unrelated patients with low number of neurofibromas.	A allele is rare.<br>No associations with diseases described, only detected by tumour sequencing in colorectal cancer (Chang et al. 2016).
rs112796669	G=0.999*<br>A=0.001*	G=0.982<br>A=0.018	G=1<br>A=0	G=0.972<br>A=0.278	G/G (0.96, 26/27)<br>G/A (0.04, 1/27): in a patient with high neurofibroma burden.	A allele is more common in NF1 patients with high tumor burden.<br>No associations with diseases described.
rs1805326	C=0.837<br>T=0.163	C=xx<br>T=xx	C=0.03<br>T=0.97	C=0<br>T=1	TT (0.97, 33/34)<br>T/C (0.03, 1/34)<br>C/C (0, 0/34): in 1 patient with 2 neurofibromas.	No associations with diseases described.

* Information according to NCBI (1000 genomes, Europe subgroup). # Refers to number of neurofibroma as defined above for the study groups. ** Information according to the genome Aggregation Database (gnomAD), European subgroup since 1000 genomes was not available. F_{NF1} represents frequency in all NF1 patients investigated in this study, F_{low} and F_{high} denotes allele frequency of NF1 patients with a low or high number of neurofibroma according to above defined criteria. °° All genotypes were not significantly different (p > 0.05) between NF1 subgroups (X^2 test) underlining significance of very rare genotypes which is commented in the table.

References from table B:

Auclair J et al. (2006) Hum Mutat 27:145-154. doi:10.1002/humu.20280
Beiner ME et al. (2006) Cancer Epidemiol Biomarkers Prev 15:1636-1640 doi:10.1158/1055-9965.EPI-06-0257
Boeckmann L et al. (2009) Genomics 19:760-769 doi:10.1097/FPC.0b013e3283307cd9
Chang YC et al. (2016) World J Gastroenterol 22:2314-2325 doi:10.3748/wjg.v22.i7.2314
Doherty JA et al. (2013) Int J Mol Epidemiol Genet 4:11-34
Doss CGP, Sethumadhavan R (2009) J Biomed Sci 16 doi:Artn 4210.1186/1423-0127-16-42
Hishida A et al. (2003) Cancer Genet Cytogenet 147:71-74 doi:10.1016/s0165-4608(03)00185-7
Jung CY et al. (2006) Cancer Epidemiol Biomarkers Prev 15:762-768 doi:10.1158/1055-9965.EPI-05-0834
Maertens O et al. (2006) Hum Mutat 27:1030-1040 doi:10.1002/humu.20389
Mik M et al. (2017) Arch Med Sci 13:1295-1302 doi:10.5114/aoms.2017.67024
Nejda N et al. (2009) Cancer Genet Cytogenet 193:71-77 doi:10.1016/j.cancergencyto.2009.04.011
Paz-y-Mino C et al. Leuk Lymphoma 44:505-508 doi:10.1080/1042819021000047038
Paz-y-Mino C et al. (2002) Cancer Genet Cytogenet 133:29-33 doi:10.1016/s0165-4608(01)00547-7
Rajaraman P et al. (2010) Neuro Oncol 12:37-48 doi:10.1093/neuonc/nop012
Roscigno RF et al. (1993) J Biol Chem 268:11222-11229
Si W et al (2019) Int J Gynecol Cancer 29:1148-1155 doi:10.1136/ijgc-2019-000368
Smolarz B et al. (2019) Pathol Oncol Res 25:1311-1317 doi:10.1007/s12253-017-0370-8
Tournier I et al. (2008) Hum Mutat 29:1412-1424 doi:10.1002/humu.20796
Zhao X et al. (2019) Gastric Cancer 22:1121-1129 doi:10.1007/s10120-019-00962-8
